# Recombinant Human Thymosin β4 (rhTβ4) Modulates the Anti-Inflammatory Responses to Alleviate Benzalkonium Chloride (BAC)-Induced Dry Eye Disease

**DOI:** 10.3390/ijms23105458

**Published:** 2022-05-13

**Authors:** Yanfang Zhai, Xiaoxiang Zheng, Yunyun Mao, Kai Li, Yanhong Liu, Yuemei Gao, Mengsu Zhao, Rui Yang, Rui Yu, Wei Chen

**Affiliations:** 1 Beijing Institute of Biotechnology, Academy of Military Medical Sciences, Beijing 100071, China; zyf_2022@163.com (Y.Z.); myy-0706@163.com (Y.M.); 13910096703@139.com (K.L.); lyhong0103@163.com (Y.L.); gym7178@126.com (Y.G.); zmsusu@126.com (M.Z.); 18515366613@163.com (R.Y.); 2 Eye Institute of Xiamen University & Fujian Provincial Key Laboratory of Ophthalmology and Visual Science, School of Medicine, Xiamen University, Xiamen 361104, China; zhengxiaoxiang11@126.com

**Keywords:** benzalkonium chloride (BAC), dry eye disease (DED), recombinant human thymosin β4 (rhTβ4), inflammation

## Abstract

Dry eye disease (DED) is a multifactorial ocular disorder that interferes with daily living and reduces quality of life. However, there is no most ideal therapeutic treatment to address all the deleterious defects of DED. The purpose of this study was to investigate the ability of recombinant human thymosin β4 (rhTβ4) to promote healing in a benzalkonium chloride (BAC)-induced mice DED model and the anti-inflammatory effects involved in that process. Eye drops consisting of 0.05% and 0.1% rhTβ4 were used for treatment of DED. Tear volume and corneal staining scores were measured after 7 days. Periodic acid-Schiff staining for gobleT cells in conjunctiva, immunohistochemical staining for CD4^+^ T cells, TUNEL assay for apoptotic positive cells in cornea and conjunctiva, qRT-PCR and ELISA assays for multiple cytokines were performed. All clinical parameters showed improvement in both the 0.05% and 0.1% rhTβ4 groups. Specifically, topical application of rhTβ4 significantly increased conjunctival gobleT cells and reduced apoptotic cells in conjunctiva. Mechanically, the rhTβ4 groups showed significantly reduced inflammatory cytokine levels and CD4^+^ T cells in conjunctiva by blocking NF-κB (nuclear factor kappa B) activation, suggesting that 0.05–0.1% rhTβ4 eye drops may be used as a potential therapeutic treatment for DED.

## 1. Introduction

Dry eye disease, also known as the xerophthalmia, is a disease of the ocular surface due to comprehensive disturbances of the natural protection in the external eye, with dry eyes as the main symptom, often accompanied by itching, burning, photophobia, blurred vision, and visual fluctuations. Multiple risk factors are related to DED, including personal (gender, age, daily habits, medication, and autoimmune diseases) and environmental (temperature and humidity) factors. The incidence of the disease ranges from 5 to 50%, with more women and elders developing serious DED [1]. Pathophysiologically, dysregulated immune-mediated inflammation involves both the ocular surface and the lacrimal gland attributed to the DED, including boosted levels of inflammatory cytokines, especially IL-1, IL-6, and TNF-α [2]. Because the causes of DED are complicated and heterogeneous, the main purpose of DED therapy lies in effective control of ocular lubrication. Symptomatic treatments to provide palliative relief include personal habits changes, dietary modifications and topical medications, depending on DED severity. Currently, immunomodulatory agents, such as Cyclosporine A (CsA) 0.05% ophthalmic emulsions, has been used to relieve inflammation on the ocular surface, but clinical limitations still remain [3]. Therefore, more universal and more effective immunomodulations as the therapeutic approach for DED have been studied.

Benzalkonium chloride (BAC), as a commonly used preservative in ophthalmic preparations, is a quaternary ammonium compound that has been reported to cause damage to both the cornea and conjunctiva in recent years [4,5,6]. Reliable dry eye models have been successfully developed in rabbits [7] and mice [8] using topical instillations of BAC. More importantly, the improvement of the tear film and the ocular surface using BAC-induced DED models would assist drug evaluation.

Thymosin β4 (Tβ4), a highly conserved 43 aa peptide with the N-terminal acetylated, is widely distributed in various tissues and cells in humans. Various biological activities are found in different fragments of Tβ4. For example, the 1–4 aa shows anti-inflammatory activities, thus preventing tissue damage and fibrosis [9]; the 1–15 aa harbors anti-apoptotic function and protects cells from damage [10], and the 17–23 aa plays a role in hair growth and wound healing [11]. These diverse physiological functions also give Tβ4 the potential for a variety of clinical applications, including injury repair of lung, liver, kidney, skin, heart, and other organs. A therapeutic effect of Tβ4 on corneal injury has been reported, demonstrated in corneal debridement and alkali injury models [12,13,14]. Clinically, several studies have reported that topical Tβ4 application relieved ocular discomfort in human and experimental DED [15,16]. Until now, the mechanisms of Tβ4 on DED treatment are not clear, especially on inflammatory regulation in injured conjunctiva.

Herein, we established a recombinant human thymosin beta-4 (rhTβ4) in modified *E. coli* [17]. rhTβ4 possessed exactly the same structure and function as the native Tβ4. In the present study, the effects of topical 0.05% and 0.1% rhTβ4 on tear film, ocular surface parameters and inflammation were demonstrated in a BAC-induced mouse model of experimental dry eye.

## 2. Results

### 2.1. Healing of Dry Eye with rhTβ4 Treatments in Mice

**Figure 1:**
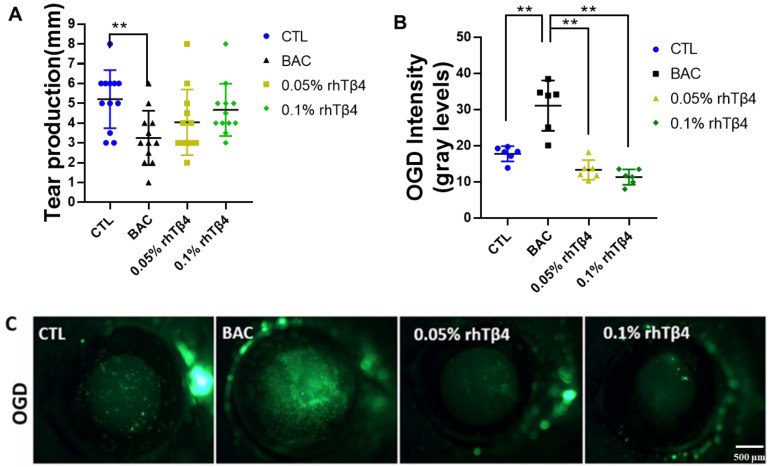
Effects of rhTβ4 on tear production and corneal barrier function in a BAC-induced DED mouse model. (**A**) Mean tear volumes (*n* = 12 eyes per group). (**B**) Corneal OGD staining scores (*n* = 6 eyes per group) and (**C**) representative images of corneal OGD staining. The rhTβ4 groups showed an improvement in all clinical parameters compared with the BAC group. ** *p* < 0.01.

Tear volumes after 7 days were tested and both 0.05% and 0.1% Tβ4 groups showed a trend of improvement compared with the BAC group (Figure 1A). OGD (dextran, oregon green) staining, which shows the integrity and permeability of corneal barrier, increased after BAC exposure. Assessments performed on the seventh day of topical drug treatment demonstrated that there was a significant treatment effect of both 0.05% and 0.1% rhTβ4 on DED when given four times a day. Analysis was performed using the Mann Whitney test for the 0.05% or 0.1% groups versus BAC control; there was a significant difference when the eyes were analyzed individually (*n* = 6, *p* = 0.0022 for the 0.05% and 0.1% rhTβ4 groups) (Figure 1B,C). These studies found that rhTβ4 was effective in promoting corneal repair in the BAC-induced DED model.

### 2.2. rhTβ4 Rescued gobleT Cells Density by Reducing Apoptosis

**Figure 2:**
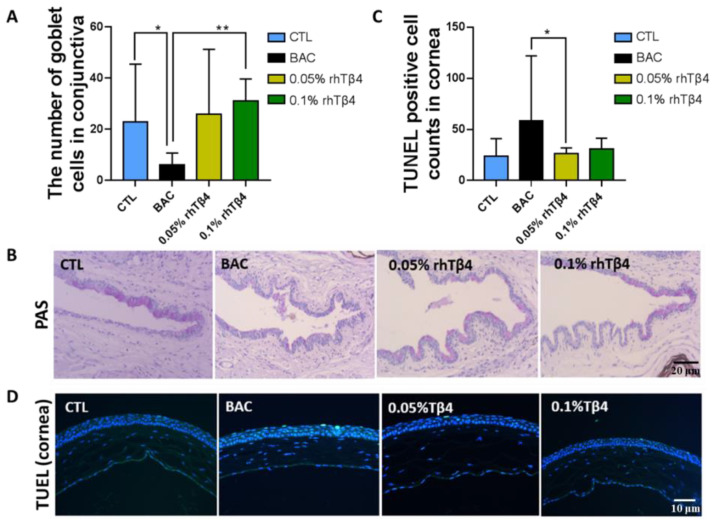
Effects of rhTβ4 on conjunctival gobleT cells numbers and corneal cell death in a BAC-induced DED mouse model. (**A**) Conjunctival gobleT cell densities and (**B**) representative images of PAS staining at day 7. Conjunctival goblet cell density was significantly increased in the rhTβ4 treatment groups. (**C**) Apoptotic positive cells in the corneal epithelium and (**D**) representative images of TUNEL staining at day 7. The rhTβ4 groups had a lower number of TUNEL positive cells in the cornea compared with the BAC group. *n* = 6 mice per group, * *p* < 0.05; ** *p* < 0.01.

At day 7, mice eyes treated with rhTβ4 were examined histologically to evaluate conjunctival and corneal integrity. Mice in the 0.1% rhTβ4 treatment group exhibited significantly higher conjunctival gobleT cell densities compared with the BAC groups (*p* = 0.0043, **) (Figure 2A). Representative images of the conjunctival sections stained with PAS are presented in Figure 2B. There was a significant decrease in the number of corneal apoptotic cells observed in the 0.05% rhTβ4 groups when compared with BAC group (*p* = 0.0433, *) (Figure 2C). Representative images of the corneal sections stained with TUNEL are presented in Figure 2D. In conjunctiva, the results had the same trend (Figure S1A,B). Of note, the higher dose (0.1%) was no more effective on dry eye at this time point. These results clearly showed a positive effect of rhTβ4 on DED.

### 2.3. rhTβ4 Modulates the Balance of Cellular Matrix Metalloproteinase Levels after BAC Injury

**Figure 3:**
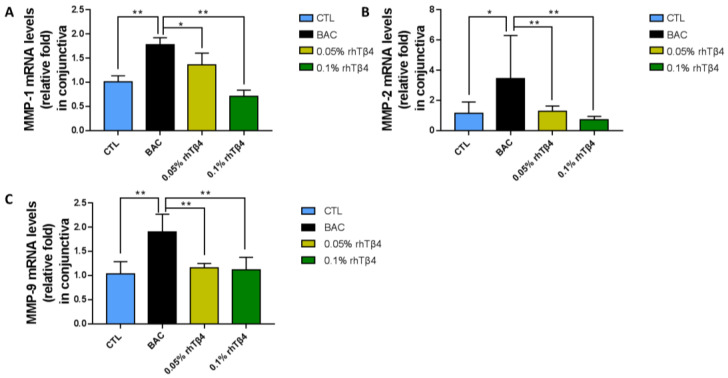
rhTβ4 effects on conjunctival MMPs expression after BAC injury. qRT-PCR analysis of MMP-1 (**A**), MMP-2 (**B**), and MMP-9 (**C**) expression at day 7 after rhTβ4 administration showed that the induction of MMP-1, MMP-2, and MMP-9 mRNA by conjunctival epithelia was inhibited by rhTβ4. *n* = 6 mice per group, * *p* < 0.05; ** *p* < 0.01.

Alterations in matrix metalloproteinase (MMPs) levels have been linked to abnormal corneal wound healing and the formation of persistent epithelial defects and chronic corneal ulcers [18,19]. We also observed that BAC injury caused significant increase in MMP-1, MMP-2, and MMP-9 mRNA in conjunctiva at day 7 (Figure 3). At the same time, qRT-PCR analysis showed that rhTβ4-treated conjunctiva had decreased gene transcript levels of MMP-1, MMP-2, and MMP-9 (Figure 3A–C). The results demonstrate that rhTβ4 may promote tissue repair by processes involving matrix remodeling.

### 2.4. rhTβ4 Modulates Conjunctival Inflammation

**Figure 4:**
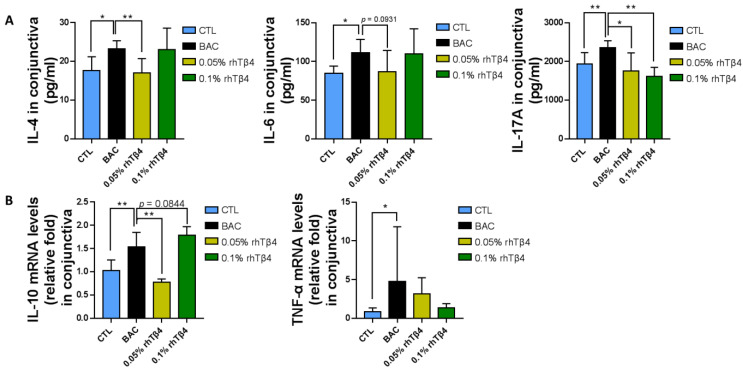
rhTβ4 decreased conjunctival pro-inflammatory cytokines’ expression after BAC injury. Conjunctiva from eyes subjected to BAC injury and treated with rhTβ4 were harvested at day 7 for ELISA and qRT-PCR analysis of a series of cytokines. ELISA quantification of IL-4, IL-6 and IL-17A (**A**) and qRT-PCR analysis of IL-10 and TNF-α (**B**) demonstrated decreased cytokines gene transcription and expression in the rhTβ4-treated eyes. *n* = 6 mice per group, * *p* < 0.05; ** *p* < 0.01.

We also examined the levels of conjunctival gene transcripts and protein levels for a series of key murine cytokines. By day 7, conjunctival IL-4, IL-6 and IL-17A protein level had decreased in rhTβ4 treatment groups, which showed non-inferiority in the 0.05% rhTβ4 group compared to 0.1% rhTβ4 group (Figure 4A) as to conjunctival IFN-γ and TNF-α (Figure S2A). Similarly, IL-10 and TNF-α expression levels were markedly reduced in the rhTβ4-treated conjunctiva (Figure 4B). In addition, nearly the same trend in IL-4, IL-6, IL-17 and TNF-α gene transcript levels was observed even though no significant difference was calculated (Figure S2B). Furthermore, histological analysis revealed that the BAC-treated group had a trend of more inflammatory CD4^+^ cell infiltration in the peripheral conjunctiva, while the rhTβ4-treated groups both showed a trend of lower counts of CD4^+^ T cells compared with the BAC group, and was not different from the 0.05% rhTβ4 and 0.1% rhTβ4 groups (Figure S3A,B). These results strongly suggest that topical rhTβ4 treatment after BAC injury down-regulates the ongoing inflammatory immune responses.

### 2.5. rhTβ4 Ameliorates NF-kB Activation

**Figure 5:**
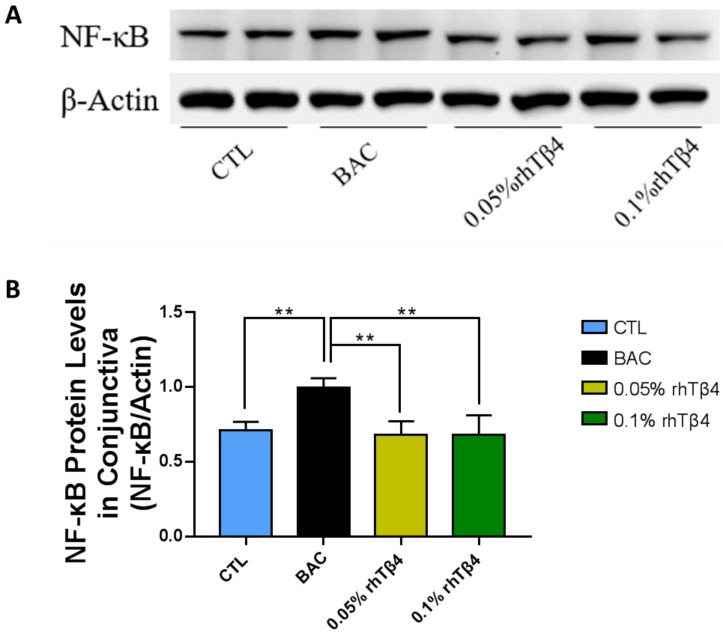
rhTβ4 suppression of NF-κB activation. Western blot analysis of NF-κB activation in conjunctiva (**A**) and the gray value were calculated and normalized to that of the corresponding β-actin (**B**). *n* = 6 mice per group, ** *p* < 0.01.

As NF-kB plays a central role in many inflammatory diseases, including ophthalmopathy, we investigated NF-kB activation in rhTβ4 treatment groups. As expected, NF-kB activation was induced in the BAC group, and was significantly reduced when treated with 0.05% and 0.1% rhTβ4 (Figure 5A,B). Notably, no greater advantage was observed in 0.1% rhTβ4 group compared to the 0.05% rhTβ4 group. Consistent with previous studies [20], these results revealed that rhTβ4 prevented the activation of NF-kB, thereby preventing the production of various proinflammatory cytokines.

## 3. Discussion

Dry eye disease is a chronic disease usually requiring long-term treatment. A variety of pharmacologic therapies for dry eye disease have limitations. For example, topical artificial tear replacement is palliative that only lubricates the surface without addressing the underlying disease process; corticosteroids, as inhibitors of multiple inflammatory processes, have considerable side effects that render them therapeutically impotent in a number of patients [2]. Therefore, drugs that offer therapeutic benefit without serious side effects have been of great interest. A previous study revealed that Tβ4 could markedly alleviate DED symptoms and accelerate wound healing, as observed in animal, as well as in human studies [21]. Improvements in tear film breakup time, tear volume and relief of eye discomfort were observed in Tβ4-treated patients [15,22,23]. However, Tβ4’s efficacy in vivo, and the mechanisms of action in BAC-induced dry eye disease, are not well understood. Herein, in the model of BAC-induced oxidative corneal injury, we proved that rhTβ4, with exactly the same structure and function as the native Tβ4, promoted the growth and survival of mice corneal epithelial cells, reduced apoptosis and inflammation, regulated the balance of cellular matrix metalloproteinases, and thus promoted wound repair and exerting a strong protective effect on damaged cornea and conjunctiva after BAC injury.

BAC has long been used as a preservative in eye medicine, and its damage to the eye surface has been the focus of ophthalmic attention. In this study, 0.075% BAC solution was used, and successfully created models of dry eye that exhibited the instability of the tear film and inflammation of the ocular surfaces in C57BL/6 mice. In this model, both 0.05% rhTβ4 and 0.1% rhTβ4 relieved symptoms and dampened inflammation almost indiscriminately, implying that a concentration of 0.05% is sufficient for protection. The effects of rhTβ4 on BAC-induced DED further recommended that a small amount of supplement of rhTβ4 to eye drops with BAC as preservative would be a potential and effective way to avoid DED.

Tβ4 is widely expressed in the human body and possesses a lot of biological activities that could promote wound repair of multiple organs, such as lung, liver, kidney, skin, and heart [11]. The mechanisms of action for injury repair seem to be the promotion of migration, downregulation of inflammatory chemokines and cytokines, inhibition of cell apoptosis, and recruitment of stem cells. Accordingly, Tβ4 could potentially be an excellent preventive and repair agent for many types of eye injuries such as neurotrophic keratopathy [24], chemical injury and corneal epithelial debridement [25]. These activities protect the eye against cytotoxic agents, accelerated healing, and reduced scar formation.

There are deficiencies in this study that need to be improved. For example, the difference between some groups was not significant, or some of the cytokine levels between 0.5% and 1% rhTβ4 groups were inconsistent. This may be due to large individual differences between animals. The sample size needs to be expanded.

Above all, the therapeutic effects of rhTβ4 on BAC-induced DED mice model revealed that rhTβ4, as an effective novel agent to promote corneal wound healing without adverse side effects, would be a major therapeutic advance in the field of ophthalmology.

## 4. Materials and Methods

### 4.1. Animals

Female C57BL/6 mice, aged 8–10 weeks (purchased from SLAC laboratory animal CO., LTD, Shanghai, China), were used in this study. The mice were quarantined and acclimatized for 3 days before the experiments in the standard IVC system in the Medical College, Xiamen University.

### 4.2. BAC and rhTβ4 Treatment

Mice were randomly assigned to five groups with six mice each. The BAC group, 0.05% rhTβ4 group, and 0.1% rhTβ4 group received a topical administration of 0.075% BAC solution (5 μL/eye). The mice were treated twice daily (9:00 a.m. and 17:00 p.m.) for 7 consecutive days. Furthermore, 0.05% rhTβ4 group and 0.1% rhTβ4 group received a topical administration of the corresponding concentrations of rhTβ4 solution (5μL/eye) four times a day (9:30 a.m., 11:30 a.m., 14:30 p.m., and 17:30 p.m.) for 7 days. The rhTβ4 used in this study was based on our patented technology (Chinese invention patent No: ZL200910135972.0 and ZL201910498316.0), which was expressed and purified from *E. coli* in our laboratory [17].

### 4.3. Measurement of Tear Production

The number of tears was measured with the phenol red thread tear test using cotton threads (Tianjin Jingming New Technology Development Co., LTD, Tianjin, China) at day 7. Animals were kept immobile, and a 1 mm portion of thread was placed on the palpebral conjunctiva at a specified point approximately 1/3 of the distance from the lateral canthus of the lower eyelid. Each eye in four groups was individually tested with the eyes open for 15 s. The red portion of the thread was measured in millimeters. After the test, eyes were closed to avoid excessive exposure and irritation of the ocular surface.

### 4.4. OGD Staining

The mice were sacrificed, and the eyes were punctured with 1 μL OGD (Thermo, Waltham, MA, USA) solution for 1 min. Then the ocular surface and palpebral conjunctiva were washed with PBS 5 times. Filter paper was used to absorb the excess dye solution. Images were taken with a fluorescence microscope, and the fluorescence intensity was analyzed by Image-Pro Plus 6.0 software.

### 4.5. Histologic Analysis and Assessment of Conjunctival Goblet Cells

At day 7, six mice in each group were sacrificed and the eyeballs were gently dissected. Six ocular specimens in each group were fixed in 10% formalin and then embedded in paraffin. The tissues sections were stained with hematoxylin and eosin (H&E) for histomorphologic analysis, and with periodic acid-Schiff (PAS) for conjunctival goblet cells. After PAS staining, the number of goblet cells in the conjunctivas was counted under a microscope (cells/high-power visual field).

### 4.6. Cryosections and Immunohistology

Cryosections of enucleated eyes were prepared and incubated with antibodies to CD4 (BD 553647, San Diego, CA, USA) and TUNEL assay (DeadEnd™ Fluorometric TUNEL System; PROMEGA G3250, Madison, WI, USA) to detect inflammatory and apoptotic cell infiltration, respectively, and counted using a fluorescent microscope.

### 4.7. ELISA

Individual conjunctiva from each group were collected and then ELISA analyses for murine IL-4 (BMS613HS), IL-6 (BMS603HS), IL-17 (BMS6001), IFN-γ (BMS606), and TNF-α (BMS607-3) were performed according to the manufacturer’s protocol. 

### 4.8. qRT-PCR

Individual conjunctiva from each group were collected and total RNA was extracted using the RNeasy Mini Kit (Qiagen 74104, Hilden, Germany) according to the manufacturer’s protocol. RT was performed with equal volumes of RNA using PrimeScript™ RT Master Mix (TaKaRa RR036A, Shiga, Japan) and then amplified using Hieff^®^ qPCR SYBR^®^ Green Master Mix (YEASEN 11201ES08, Shanghai, China). The murine primer sequences used for qRT-PCR are shown in Table 1.

### 4.9. Western Blotting

Proteins of the conjunctiva from each group were extracted with 200 μL cold RIPA buffer. The tissue was broken by ultrasound and the supernatant was transferred to a clean EP tube after high-speed centrifugation. Equal amounts of proteins of the cell lysates were subjected to electrophoresis on SDS-PAGE and then electrophoretically transferred to PVDF membranes. After 1 h blocking in 2% BSA, the membranes were incubated with primary antibodies for NF-κB (CST 8242T) and β-actin (Sigma A3854, St. Louise, MO, USA) as a loading control. After three washes with Tris-buffered saline containing 0.05% Tween-20 for 10 min each, the membranes were incubated with goat anti-rabbit IgG (Abcam 31460, Cambridge, UK) for 1 h. Specific bands were visualized by enhanced chemiluminescence reagents and recorded on film.

### 4.10. Statistical Analysis

Results were expressed as means (SE). Comparisons among the groups were performed by nonparametric comparisons (Mann-Whitney test) with significance set at * *p* < 0.05; ** *p* < 0.01.

## 5. Conclusions

Using 0.075% BAC to establish a DED mice model, the therapeutic effects of 0.05% rhTβ4 and 0.1% rhTβ4 were investigated. The results are summarized as follows.

(1) BAC treatment significantly reduced tear volume and gobleT cells, and elevated cell apoptosis and inflammation compared with healthy controls.

(2) rhTβ4 treatment promoted the growth and survival of mice corneal epithelial cells, decreased apoptosis and inflammation, and balanced the level of cellular matrix metalloproteinases, thus promoting corneal and conjunctival wound repair and overcoming the side effect of BAC.

(3) rhTβ4 at 0.05% was able to provide a protective effect in the BAC-induced DED model, implying that a tiny amount of rhTβ4 would be a useful additive to eye drops containing BAC as a preservative in order to avoid DED.

Consequently, topical administration of rhTβ4 to BAC-preserved ocular medications is a promising therapeutic strategy to alleviate DED.

## Figures and Tables

**Table 1:** Murine primer sequences used for qRT-PCR.

Gene	Sense Primer	Antisense Primer	Length, bp
MMP-1	CCGCTGCTCTCAACCATTTCCT	AGGCAGACCGCAATGGATGAAC	150
MMP-2	CCTCTGCTGCCTCTTGACCTCT	CACACCACACCTTGCCATCGT	158
MMP-9	CAATCCTTGCAATGTGGATG	AGTAAGGAAGGGGCCCTGTA	130
IL-4	CGCCTGCTCACTCTCATGGAAC	CATCTCGCCTGCCTCCTCACTT	140
IL-6	GCTGATGCTGCCTGTTGTCTAA	AAGTGGGAGTTGGTGGGTAAGG	164
IL-10	ACCAATCACGGCTCAGTTCTCC	CTGCTCCACTGCCTTGCTCTT	200
IL-17A	CGCAATGAAGACCCTGATAGAT	CTCTTGCTGGATGAGAACAGAA	123
TNF-α	AGGCTCAGGATGTGGAGTGTGA	TTGACGGCAGAGAGGAGGTTGA	303
β-actin	CCTAAGGCCAACCGTGAAAAG	AGGCATACAGGGACAGCACAG	100

## Data Availability

Not applicable.

## Supplementary Materials

The following supporting information can be downloaded at: https://www.mdpi.com/article/10.3390/ijms23105458/s1.
