# Supplementary material for: Recombinant Human Thymosin β4 (rhTβ4) Modulates the Anti-Inflammatory Responses to Alleviate Benzalkonium Chloride (BAC)-Induced Dry Eye Disease
Source: Int J Mol Sci. 2022 May 13;23(10):5458. doi: 10.3390/ijms23105458 (PMC9141377; doi:10.3390/ijms23105458)
Supplement: Supplementary file 1 [file ijms-23-05458-s001.zip › ijms-1677417-SI.pdf]

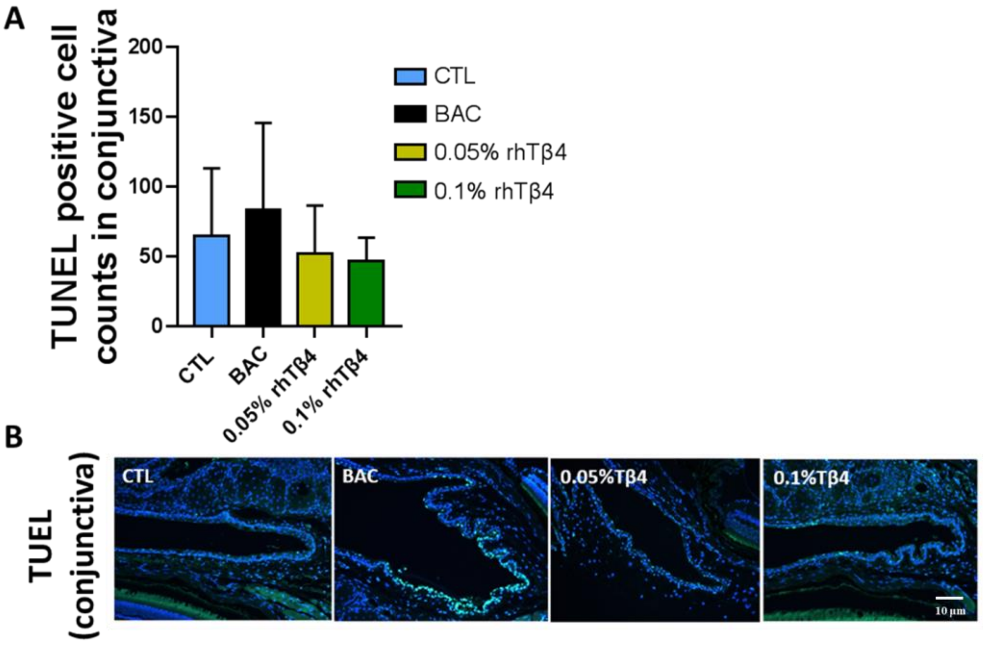

Figure S1. Effects of rhTβ4 on conjunctival cell death in BAC-induced DED mouse model. (A) Apoptotic positive cells in the conjunctiva and (B) representative images of TUNEL staining at day 7. The rhTβ4 groups had a lower number of TUNEL positive cells in the conjunctiva. n=6 mice per group. Related to Figure 2.

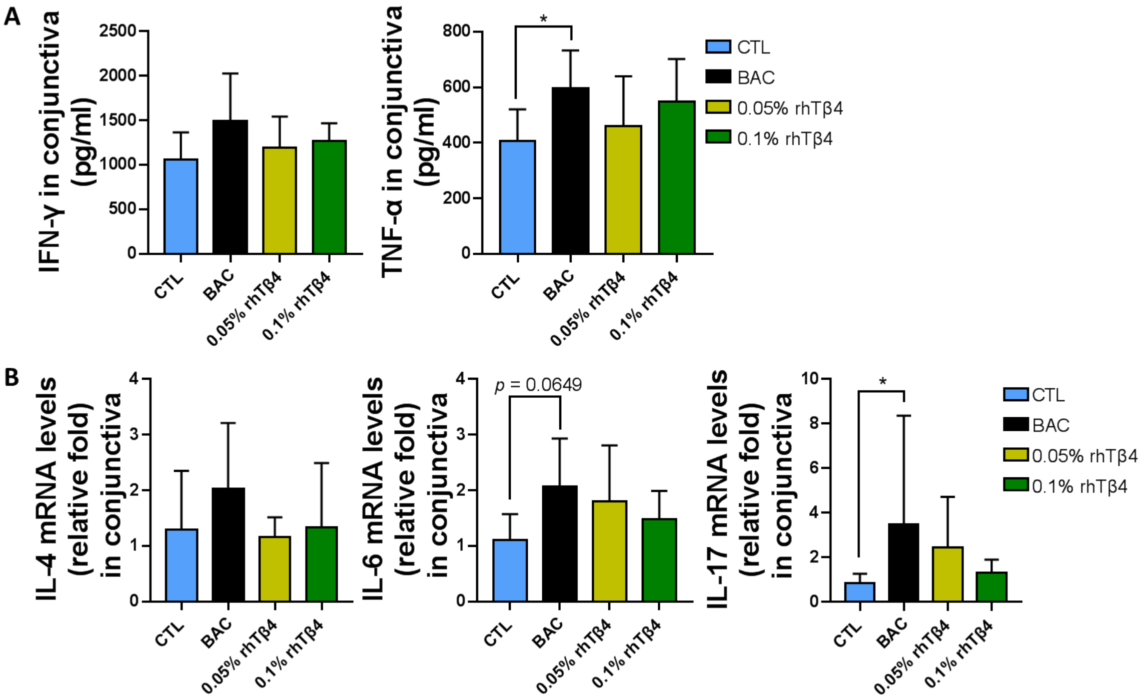

Figure S2. rhTβ4 decreased conjunctival pro-inflammatory cytokines' expression after BAC injury. ELISA quantification of IFN-γ and TNF-α (A) and qRT-PCR analysis of IL-4, IL-6, and IL-17 (B) demonstrated decreased cytokines gene transcription and expression in the rhTβ4-treated eyes. n=6 mice per group, * $p < 0.05$. Related to Figure 4.

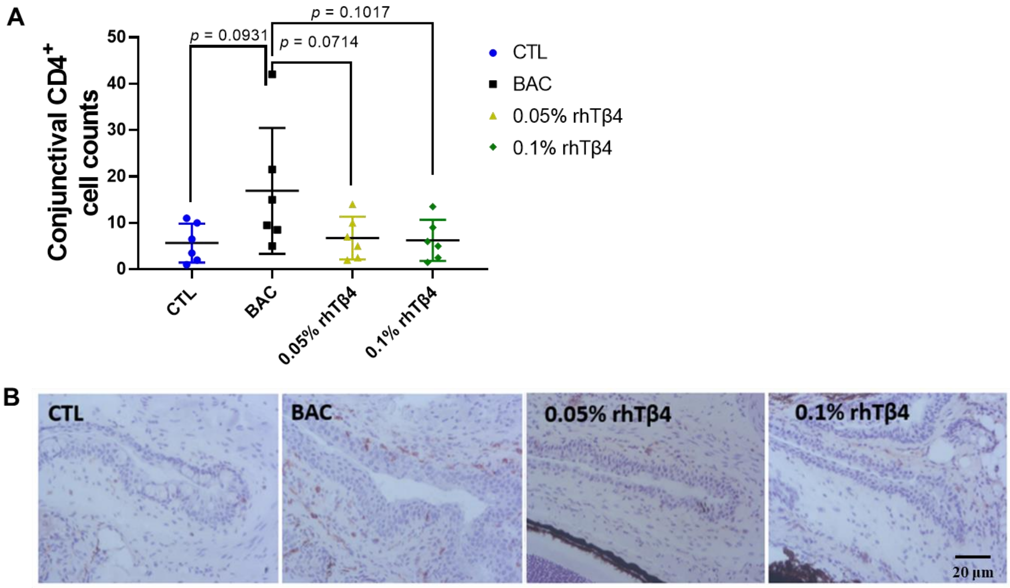

Figure S3. Analysis of degrees of CD4+ T cells infiltration after rhTβ4 treatment. Conjunctival CD4+ cells counts (A) and representative images (B) showed less inflammatory infiltration in peripheral conjunctival stroma after rhTβ4 treatme
